# Seroepidemiology of *Sarcocystis neurona* and *Neospora hughesi* infections in domestic donkeys (*Equus asinus*) in Durango, Mexico

**DOI:** 10.1051/parasite/2017030

**Published:** 2017-07-21

**Authors:** Cosme Alvarado-Esquivel, Daniel K. Howe, Michelle R. Yeargan, Domingo Alvarado-Esquivel, José Alfredo Zamarripa-Barboza, Jitender P. Dubey

**Affiliations:** 1 Biomedical Research Laboratory, Faculty of Medicine and Nutrition, Juárez University of Durango State. Avenida Universidad S/N 34000 Durango Mexico; 2 Department of Veterinary Science, M. H. Gluck Equine Research Center, University of Kentucky Lexington Kentucky 40546-0099 USA; 3 Healthcare Center No. 1 “Dr. Carlos León de la Peña”. Secretary of Health, Boulevard de la Juventud S/N 34000 Durango Mexico; 4 United States Department of Agriculture, Agricultural Research Service, Beltsville Agricultural Research Center, Animal Parasitic Diseases Laboratory, Building 1001 Beltsville Maryland 20705-2350 USA

**Keywords:** *Sarcocystis neurona*, *Neospora hughesi*, Seroprevalence, Donkeys, Cross-sectional study, Mexico

## Abstract

There is currently no information regarding *Sarcocystis neurona* and *Neospora hughesi* infections in donkeys in Mexico. Here, we determined the presence of antibodies against *S. neurona* and *N. hughesi* in donkeys in the northern Mexican state of Durango. Serum samples of 239 domestic donkeys (*Equus asinus*) were assayed for *S. neurona* and *N. hughesi* antibodies using home-made enzyme-linked immunoassays; six (2.5%) of the 239 donkeys tested seropositive for *S. neurona*. The seroprevalence of *S. neurona* infection was comparable among donkeys regardless of their origin, health status, or sex. Multivariate analysis showed that seropositivity to *S. neurona* was associated with increased age (OR = 2.95; 95% CI: 1.11–7.82; *p* = 0.02). Antibodies to *N. hughesi* were found in two (0.8%) of the 239 donkeys. Both exposed donkeys were healthy, 3- and 6-year-old females. This is the first evidence of *S. neurona* and *N. hughesi* infections in donkeys in Mexico.

## Abstract (fr)

Il n’y a actuellement aucune information concernant les infections par *Sarcocystis neurona* et *Neospora hughesi* chez les ânes au Mexique. Dans cet article, nous avons déterminé la présence d’anticorps contre *S. neurona* et *N. hughesi* chez les ânes dans l’État de Durango au nord du Mexique. Des échantillons de sérum de 239 ânes domestiques (*Equus asinus*) ont été testés pour les anticorps de *S. neurona* et *N. hughesi* à l’aide d’immunoessais enzymatiques faits maison; six (2.5 %) des 239 ânes ont été testés séropositifs pour *S. neurona*. La séroprévalence de l’infection par *S. neurona* était comparable chez les ânes, quels que soient leur origine, leur état de santé ou leur sexe. L’analyse multivariée a montré que la séropositivité à *S. neurona* était associée à l’âge (OR = 2.95 ; IC à 95 % : 1.11-7.82 ; *p* = 0.02). Les anticorps contre *N. hughesi* ont été trouvés chez 2 (0.8 %) des 239 ânes. Les deux ânes positifs étaient des femelles de 3 à 6 ans en bonne santé. Ceci est la première preuve d’infections à *S. neurona* et *N. hughesi* chez les ânes au Mexique.

## Introduction

The apicomplexan protozoa *Sarcocystis neurona* (*S. neurona*) and *Neospora hughesi* (*N. hughesi*) are the etiological agents of equine protozoal myeloencephalitis, which is an important neurological disease of horses in the Americas [3, 4]. Additionally, *Neospora* spp. can cause abortion in horses. Although *S. neurona* and *Neospora* spp. can cause disease in equids other than horses, there are no reports of clinical disease in donkeys. There is scarce information regarding *S. neurona* and *N. hughesi* infections outside of the United States [3, 4]. We recently reported the first seroprevalence of these infections in horses in Durango, Mexico [16]. However, we are not aware of any study of these infections in donkeys (*Equus asinus*) in Mexico. Here we determined the seroprevalences of *S. neurona* and *N. hughesi* infections in donkeys in the northern Mexican state of Durango because there are no reports of these infections in donkeys.

## Materials and methods

### Study design, donkeys surveyed, and serological examination

We performed a cross-sectional study using serum samples from a previous *Toxoplasma gondii* serosurvey of 239 domestic donkeys in Durango, Mexico [1]. As a strategy to enroll donkeys in the study, we obtained permission to sample donkeys in four equid gathering premises (trade centers) in the municipality of Durango, Mexico. These premises trade donkeys for slaughter in abattoirs outside Durango State. All donkeys were mixed breed. A veterinarian obtained the clinical data for the donkeys. Of the 239 donkeys examined clinically, 193 were healthy, one was malnourished, one had an abdominal mass, and 44 had dermal sores. Donkeys were 0.2–12 years old, and included 170 (71.1%) females and 69 (28.9%) males. According to the donkeys’ owners, all donkeys were kept on pasture.

Serum samples were evaluated for antibodies against *S. neurona* and *N. hughesi* using the rSnSAG2/4/3 [15] and the rNhSAG1 [10] enzyme-linked immunosorbent assays (ELISAs), respectively. Antibodies were detected using goat anti-horse IgG (Jackson ImmunoResearch Laboratories, Inc., West Grove, PA, USA) diluted 1:10,000. The positive control serum for *S. neurona* was from a horse with equine protozoal myeloencephalitis that was confirmed histologically [9]. The positive control serum for *N. hughesi* was from a mare with confirmed *N. hughesi* transplacental passage to the foal (kindly provided by Dr. Nicola Pusterla, University of California-Davis, USA). The negative control serum for both ELISAs was a pre-infection sample collected from a weanling used in a prior infection trial. Percent positivity (PP) values were calculated using the optical densities obtained from the test sample and the positive and negative control samples, as described previously [9]. PP cut-off values of 10% and 20% were used for the *S. neurona* and *N. hughesi* ELISAs, respectively. Western blot analysis using whole-parasite antigen was conducted with all ELISA-positive samples to confirm the presence of antibodies.

### Statistical analysis

For statistical analysis, SPSS 15.0 software (SPSS Inc., Chicago, IL, USA) and the Fisher exact test were used for comparison of the frequencies among groups. The association between the donkeys’ characteristics and *S. neurona* and *N. hughesi* seropositivities was analyzed by stepwise regression analysis using the backwards elimination method. The dependent variable was seropositivity to *S. neurona*. Independent variables included in the regression analysis were only those with *p* < 0.35 obtained in the bivariate analysis: age, sex, and health status. We calculated the odds ratio (OR) and 95% confidence interval (CI), and a *p* value of < 0.05 was considered statistically significant. The Hosmer-Lemeshow goodness of fit test to assess the fitness of the regression model was used.

## Results

Antibodies to *S. neurona* were found in 6 (2.5%) of the 239 donkeys. A correlation between seropositivity to *S. neurona* and donkeys’ characteristics is shown in Table 1. Seroprevalence of *S. neurona* infection was comparable (*p* = 0.65) in donkeys from the valleys region (3.2%) and in those from the mountainous region (2.3%) (Table 1). Seropositive donkeys were found in only one (25%) of the four gathering premises studied, and they came from two (66.7%) of the 3 municipalities.

**Table 1.** General data for the 239 donkeys studied and seroprevalence of *Sarcocystis neurona* infection.

	Donkeys tested	Seroprevalence of S. neurona infection		
Characteristics	No.	No.	%	*p-*value
Age (years)				
< 1	11	0	0	0.008
1–5	135	0	0	
> 5	93	6	6.5	
Sex				
Male	69	0	0	0.18
Female	170	6	3.5	
Health status				
Ill	46	2	4.3	0.32
Healthy	193	4	2.1	
Region				
Mountains	177	4	2.3	0.65
Valleys	62	2	3.2	
Municipality				
Durango	62	2	3.2	0.86
Mezquital	172	4	2.3	
San Dimas	5	0	0	
Trade center				
1	170	6	3.5	0.47
2	5	0	0	
3	38	0	0	
4	26	0	0	

The seroprevalence of *S. neurona* infection was comparable among donkeys regardless of their health status or sex (Table 1). Seropositivity to *S. neurona* was observed only in donkeys aged 6–12 years old. Thus, the seroprevalence of *S. neurona* was significantly higher in donkeys > 5 years old than in younger donkeys (*p* = 0.008). The variables age, sex, and health status showed *p* values lower than 0.35 in the bivariate analysis and were included in the regression analysis. Multivariate analysis showed that seropositivity to *S. neurona* was associated with increased age (OR = 2.95; 95% CI: 1.11–7.82; *p* = 0.02). However, the other two characteristics of donkeys (sex and health status) were not associated with *S. neurona* seropositivity by multivariate analysis. The result of the Hosmer-Lemeshow test was 5 (*p* = 0.08), indicating an acceptable fit of our regression model.

Antibodies to *N. hughesi* were found in 2 (0.8%) of the 239 donkeys. One seropositive case was from the valleys region and the second from the mountainous region. Donkeys seropositive to *N. hughesi* were found in only 1 (25%) of the 4 gathering premises, and they came from the municipalities of Durango and Mezquital. Both donkeys seropositive for *N. hughesi* were healthy females, 3 and 6 years old.

## Discussion

The seroepidemiology of infection with *S. neurona* and *N. hughesi* in equids other than horses has received little attention. The 2.5% seroprevalence of *S. neurona* infection found in donkeys in Durango was unexpected since a 48.5% seroprevalence of this infection was found in horses in the same Durango State [16]. Opossums of the *Didelphis virginiana* species, one of the definitive hosts of *S. neurona* [3], are present widely in Durango State as shown in a previous study of *T. gondii* infection [5]. It is unclear why donkeys had a lower seroprevalence of *S. neurona* infection than horses. The same immunoassay to detect anti-*S. neurona* antibodies was used in both studies. This assay is based on three parasite surface proteins that are highly immunogenic, and it is likely that the antigens elicit robust antibody responses in donkeys similar to what is observed in all other animals infected or immunized with *S. neurona*. It is possible that horses were in greater contact with opossums than donkeys. Opossums are present widely in urban Durango. We are not aware of any reports regarding differences in densities of opossums in urban and rural areas. However, it was suggested that urban areas provide more resources and may be beneficial to opossum populations since opossums living within the city limits had a larger average body mass than those in rural areas [14]. Interestingly, urban horses had a higher seroprevalence of *S. neurona* than rural horses [16]. Donkeys in the present study were from rural areas of Durango State. Donkeys studied were pastured on large rural lands and the likelihood of contact with opossum feces was perhaps low. Horses fed with grains and crops had a significantly higher seroprevalence of *S. neurona* than horses fed with grass [16]. We searched for factors associated with *S. neurona* in the donkeys studied. Multivariate analysis showed that seropositivity to *S. neurona* was associated with increased age. Donkeys older than 5 years had the highest seroprevalence of *S. neurona* infection. This finding is consistent with a previous observation in horses. In a recent study in the United States, age > 5 years in horses was associated with *S. neurona* seropositivity [11]. As for horses, the likelihood of infection with *S. neurona* increases with age.

There are few reports on the seroprevalence of *S. neurona* in equids other than horses. We are aware of only two reports of *S. neurona* prevalence in donkeys. Sera of 18 donkeys from Ohio, USA were analyzed for antibodies to *S. neurona* using an immunoblot, and 11 (61.1%) of them were positive [13]. Sera of 333 donkeys from four states in Brazil were tested for antibodies to *S. neurona* by using the indirect fluorescent antibody test (IFAT, cut-off 1:40) and direct agglutination tests (SAT, cut-off 1:50). Ten (3.0%) were seropositive by IFAT and 69 (21.0%) were positive by SAT [7]. However, comparison of these seroprevalences should be interpreted with care since different laboratory methods were used to detect anti-*S. neurona* antibodies among the studies. In both IFAT and SAT, whole merozoites are used as antigen, whereas in the present study, ELISA based on recombinant antigens was used.

With respect to *N. hughesi* infection, donkeys had an unexpectedly low (0.8%) seroprevalence of this infection. This seroprevalence is comparable with a 2% seroprevalence of *Neospora* reported in 333 donkeys from the north-eastern region of Brazil using IFAT (cut-off 1:40) [7]. In another study in Brazil, researchers found antibodies to *Neospora* in 2 of 500 donkeys studied using IFAT (cut-off 1:100) [6]. Antibodies to *Neospora* were also reported in 11 (19.7%) of 56 donkeys from Colombia by using Dot-ELISA [2], in 52 (52%) of 100 donkeys from Iran by the *Neospora* agglutination test (NAT, cut-off 1:80) [8], and 28 (11.8%) of 238 donkeys from Italy by using competitive inhibition ELISA [12]. Again, comparison of seroprevalences among the studies should be interpreted cautiously because of different laboratory methods used to detect anti-*Neospora* antibodies. Additionally, reports from Brazil, Colombia, Iran, and Italy used antigen of *Neospora caninum,* whereas in the present study, antigens from *N. hughesi* were used. Currently, there are two species of *Neospora: N. caninum* with a wide host range, and dogs (*Canis domesticus*), coyotes (*Canis latrans*), and wolves (*Canis lupus*) as definitive hosts, and *N. hughesi* with horses as intermediate hosts, and unknown definitive hosts [4].

## Conclusions

We conclude that seroprevalences of *S. neurona* and *N. hughesi* infections are low in donkeys in Durango, Mexico. This is the first study that provides serological evidence of *S. neurona* and *N. hughesi* infections in donkeys in Mexico.

## Conflict of interest

The authors declare that they have no conflict of interest.
